# Correlative Light Electron Microscopy: Connecting Synaptic Structure and Function

**DOI:** 10.3389/fnsyn.2016.00028

**Published:** 2016-08-23

**Authors:** Isabell Begemann, Milos Galic

**Affiliations:** ^1^DFG Cluster of Excellence ‘Cells in Motion’, (EXC 1003), University of Muenster, MuensterGermany; ^2^Institute of Medical Physics and Biophysics, University Hospital Münster, University of Muenster, MuensterGermany

**Keywords:** correlative light electron microscopy, CLEM, synapse, neuron, fluorescence microscopy, electron microscopy, TEM, SEM

## Abstract

Many core paradigms of contemporary neuroscience are based on information obtained by electron or light microscopy. Intriguingly, these two imaging techniques are often viewed as complementary, yet separate entities. Recent technological advancements in microscopy techniques, labeling tools, and fixation or preparation procedures have fueled the development of a series of hybrid approaches that allow correlating functional fluorescence microscopy data and ultrastructural information from electron micrographs from a singular biological event. As correlative light electron microscopy (CLEM) approaches become increasingly accessible, long-standing neurobiological questions regarding structure-function relation are being revisited. In this review, we will survey what developments in electron and light microscopy have spurred the advent of correlative approaches, highlight the most relevant CLEM techniques that are currently available, and discuss its potential and limitations with respect to neuronal and synapse-specific applications.

## Introduction

Neurons transmit the majority of trans-cellular signals via synaptic contacts. To correctly and reliably respond to various stimuli (e.g., different input frequencies), each synapse hosts an elaborate machinery to regulate signal transmission in a context-dependent manner. On the molecular level, synaptic transmission in its most simple form relies on fusion of ~40 nm large vesicles at the presynaptic site, diffusion of released neuro-transmitters across the 30 nm wide synaptic cleft, and subsequent ligand-specific activation of receptors at the postsynaptic site. Considering the small size of this signaling unit, it is not surprising that major advancements in understanding synaptic function have been closely correlated with progress in imaging technology. For instance, the notion that communication across synaptic clefts relies on chemical signals would not have been possible without detailed information from electron micrographs on synapse ultrastructure (Derobertis and Bennett, 1955; Pappas and Bennett, 1966) and vesicle dynamics (Heuser and Reese, 1973). Likewise, our understanding about the function of individual synaptic proteins has substantially advanced with the introduction of ion and protein markers (Shimomura et al., 1962; Tsien, 1981; Heim et al., 1994) for fluorescence light microscopy. Intriguingly, and despite the fact that today’s neuroscience is building up on information obtained with electron and light microscopy, strategies to combine these two approaches only recently have started gaining traction.

The emergence of such dual approaches, termed correlative light electron microscopy (CLEM), from a sparsely known branch of imaging to center stage can be linked to a series of landmark papers from the nineties (Deerinck et al., 1994; Svitkina et al., 1995). Since then, fueled by advances in imaging and labeling techniques, publications using CLEM approaches have steadily been rising. In this review, we will revisit what developments have incited the advent of correlative imaging approaches, and highlight the most relevant dual microscopy techniques that are currently available. We begin by giving an overview of electron and light-based imaging methods that can be used for CLEM, followed by a section describing how individual approaches can be successfully combined to create correlative approaches. Next, we will reflect on technical concerns that need to be taken care of when designing dual imaging experiments, before closing with a discussion on current limitations and future challenges associated with CLEM.

## Electron and Light-Based Techniques Used For CLEM

CLEM describes a continuously growing number of procedures that allow merging electron and light-based images from the same object. Thus, at least in theory, each existing electron and light microscopy technique where ultrastructure remains intact could be paired to generate a CLEM image. To illustrate this combinatorial potential, and to expound existing limitations, we begin this section by discussing electron and light microscopy techniques suitable for correlative approaches, before proceeding to examples where CLEM was successful applied to study neuronal and synaptic function.

### Electron Microscopy–Visualizing the Ultrastructure

Electron micrographs in CLEM rely to a large extent on transmission electron microscopy (TEM) and scanning electron microscopy (SEM). TEM, which enables visualization of 50–100 nm thick cross-sections of samples with a resolution of down to a few Angstrom (Pierce and Buseck, 1974; Ruska, 1987; Erni et al., 2009), was critically involved in the detailed characterization of synaptic structures (Gray, 1959; Pappas and Bennett, 1966; Fifkova and Delay, 1982; Landis and Reese, 1983; Watanabe et al., 2013, 2014), and has helped to advance our understanding on age and disease-dependent changes in synaptic properties (DeKosky and Scheff, 1990; Navlakha et al., 2013).

Contrary to TEM, where electron shadows are used to create the image, SEM-based strategies utilizes the interaction of electrons with molecules in the sample to recreate the image. One commonly applied SEM strategy, where secondary electrons are used to determine the surface topography of objects with a precision of ~1 nm (Vernon-Parry, 2000), was central for investigating surface features such as the morphology of neuromuscular junctions (Desaki and Uehara, 1981) as well as structural reorganization during exploratory dendritic filopodia formation in hippocampal neurons (Galic et al., 2014). In addition, back-scattered electrons created by interaction with heavy elements (i.e., high atomic number) in the uncoated sample can also be used to render images comparable to transmission electron micrographs of ultrathin sections, an approach that is of particular relevance for embedded sections on an electron-opaque surface or samples that are investigated ‘*en block*’ (Briggman et al., 2011).

A single cross-section, or the surface of the sample, is lacking information on the three-dimensional organization within the biological specimen. Here, cryo-fracture, where frozen samples are broken to expose cell structures along the fracture line (Castejon and Caraballo, 1980; Castejon, 1996), or unroofing, where intracellular structures are uncovered by brief bursts of ultrasound (Lang, 2003), can be used together with deep-etching (Heuser and Salpeter, 1979) to gain insights into subcellular organization. While useful, these strategies lack the capability for systematic three-dimensional sample reconstruction that can be achieved with serial sectioning, where successive ultrathin slices are imaged. Early attempts of serial sectioning reach back to the late 60s (De Rosier and Klug, 1968), and have since then continuously been used to investigate neuronal networks (White et al., 1986) and synaptic connection (Mishchenko et al., 2010). In modern serial section TEM (ssTEM), sections of 60 ± 20 nm are cut with an ultra-microtome and placed manually on a metal support grid (Harris et al., 2006). More recently, automated tape-collecting ultra-microtome SEM (ATUM-SEM) has emerged as a powerful alternative (Hayworth et al., 2006; Kasthuri et al., 2007, 2015; Terasaki et al., 2013; Morgan et al., 2016). Here, sections of 30 nm are cut by an ultra-microtome and collected from the water bath using a conveyor-belt like support tape. As the support tape is electron-opaque, a finely focused SEM beam is applied to raster the surface of the sample and backscattered electrons are used to reconstruct the image. Thus, compared to ssTEM, ATUM-based strategies not only allow thinner sectioning and rapid imaging of larger areas but also substantially reduce errors associated with manual sample handling (Hayworth et al., 2014). Alternatively, an embedded tissue block can also be sectioned directly within the SEM vacuum chamber, either using a diamond knife (serial block-face SEM, SBEM; Leighton, 1981; Denk and Horstmann, 2004) or by milling with a focused ion-beam (FIB-SEM; Watkins et al., 1986; Knott et al., 2008). In the latter, a scanning electron microscope is used to image the surface of the embedded sample, while a high current focused ion beam continuously slices off sections perpendicular to the SEM axis, thus allowing 3D reconstruction of the sample. Compared to diamond-based sectioning, FIB-SEM is thus not only faster but also permits sectioning samples with a step size in the single nm range (Knott et al., 2008; Merchan-Perez et al., 2009; Lehmann et al., 2014).

Another strategy for detailed three-dimensional reconstructions relies on electron tomography (ET). In this approach, a tilt series (usually ranging from -60° to +60°) of two-dimensional images is generated to reconstruct the three-dimensional shape of an object within the single slice (Crowther et al., 1970; Hoppe et al., 1974; Penczek, 2010; Ercius et al., 2015). ET was successfully used to resolve ultrastructural features of the presynapse (Perkins et al., 2015) and synaptic vesicle populations in saccular hair cells (Lenzi et al., 1999). Yet, while ET is suitable for atomic-resolution, radiation damage, and limitations in interpretability for thicker sections need to be considered when preparing the sample (Tocheva et al., 2010).

Finally, we would like to note that further electron-based imaging strategies exist (e.g., STEM (Crewe et al., 1970; Engel, 1978), tSEM (Kuwajima et al., 2013)). While not commonly used in CLEM studies, it is plausible to envision that some of these additional types of electron microscopy may be beneficial for a particular question. For readers interested in learning more on electron microscopy techniques, we recommend reading one of the many excellent reviews available on this exciting topic (Briggman and Bock, 2012; Knott and Genoud, 2013; Miranda et al., 2015; Perkins et al., 2015).

### Light Microscopy–Monitoring and Manipulating Cell Function

While the electron microscopy techniques described above are well suited to investigate ultrastructural features with high axial resolution, they lack spatio-temporal information available with light microscopy. To increase penetration depth and reduce background compared to whole field illumination (Epi), CLEM studies frequently rely on confocal microscopy, where a pinhole is used to reduce out-of-focus light (Minsky, 1988), light sheet microscopy, where a light beam perpendicular to the objective illuminates only the focal plane (Engelbrecht and Stelzer, 2006; Keller et al., 2008), or two-photon microscopy, where simultaneous absorption of two photons is used for spatially controlled illumination (Denk et al., 1990). Resolution in light and electron microscopy is based on numerical aperture and wavelength. However, unlike in electron microscopy, where resolution is limited by the spot size (SEM) or the grain of the detector (TEM), the limiting factors in light microscopy is the wavelength (Rayleigh, 1879; Abbe, 1883). To reach beyond the diffraction limit, several approaches have been introduced over the last decade. Among the most prominent super-resolution microscopy techniques used in CLEM studies are stimulated emission depletion microscopy (STED), where a depletion laser limits the width of the emitting light source (Hell and Wichmann, 1994; Klar et al., 2001), and stochastic techniques, such as fluorescent photo-activation localization microscopy (PALM; Betzig and Chichester, 1993; Betzig et al., 2006) or stochastic optical reconstruction microscopy (STORM; Rust et al., 2006), where light emitted from sequentially activated fluorophores is fitted to determine its precise localization. As before, we would like to note that additional light-based approaches [e.g., total internal reflection fluorescence microscopy (Axelrod, 1981) or structured illumination microscopy (Neil et al., 1997)] can be used in CLEM approaches. Readers interested to learn more about fluorescence microscopy techniques, we refer to one of the many excellent reviews focused on these topics (Lichtman and Conchello, 2005; Combs, 2010; Huang et al., 2010).

Fluorescence light microscopy not only allows to visualize cells within neuronal circuits (Livet et al., 2007), but a continuously growing number of molecular probes and genetically encoded markers also provide tools to study a variety of neuronal and synaptic properties. Parameters that can be analyzed include among others subcellular protein localization (Chalfie et al., 1994; Heim et al., 1994; Kilgore et al., 2013), protein activity (Adams et al., 1991), ion dynamics (Tsien, 1980, 1981; Tsien et al., 1982; Minta and Tsien, 1989; Nakai et al., 2001), pH (Tanasugarn et al., 1984; Miesenbock et al., 1998), membrane potential or voltage (Davila et al., 1973; Siegel and Isacoff, 1997; Zochowski et al., 2000), or lipid species (Stauffer et al., 1998). Intriguingly, light is also suitable to actively manipulate the biological sample in a precise spatio-temporal manner. One commonly used approach relies on light-activation of caged substrates. Relevant for neurobiology, strategies for uncaging calcium (Ellisdavies and Kaplan, 1994), IP3 (Wang and Augustine, 1995), and various neurotransmitters (Ellis-Davies, 2011) have been realized. More recently, light has also been used to regulate protein function (Cao et al., 2008; Tischer and Weiner, 2014). This approach, coined optogenetics, has provided among others tools for controlling neuronal and synaptic activity with unprecedented spatio-temporal precision (Boyden et al., 2005; Fenno et al., 2011; Rost et al., 2015). Finally, it is worth mentioning that even physical cell parameters, such as shape or membrane tension, can be altered by light in living samples, using for instance optical tweezers (Ashkin, 1970; Zhang and Liu, 2008). In summary, when combined with electron microscopy, these light-based approaches allow precisely altering a specific parameter while monitoring the cellular responses followed by analysis of the corresponding ultrastructural features.

### Correlative Light and Electron Microscopy in Neuroscience

Although the potential of combining ultrastructural information with functional studies was early noticed, first attempts to combine these microscopy techniques were limited to depicting separately prepared and imaged biological samples (Porter et al., 1945). Experiments where fluorescence and EM images of subcellular structures from the same cell were aligned started appearing in the 1970s (Hollander, 1970; Nakai and Iwashita, 1976), and have since then been applied to investigate a variety of neurobiological questions.

**FIGURE 1:**
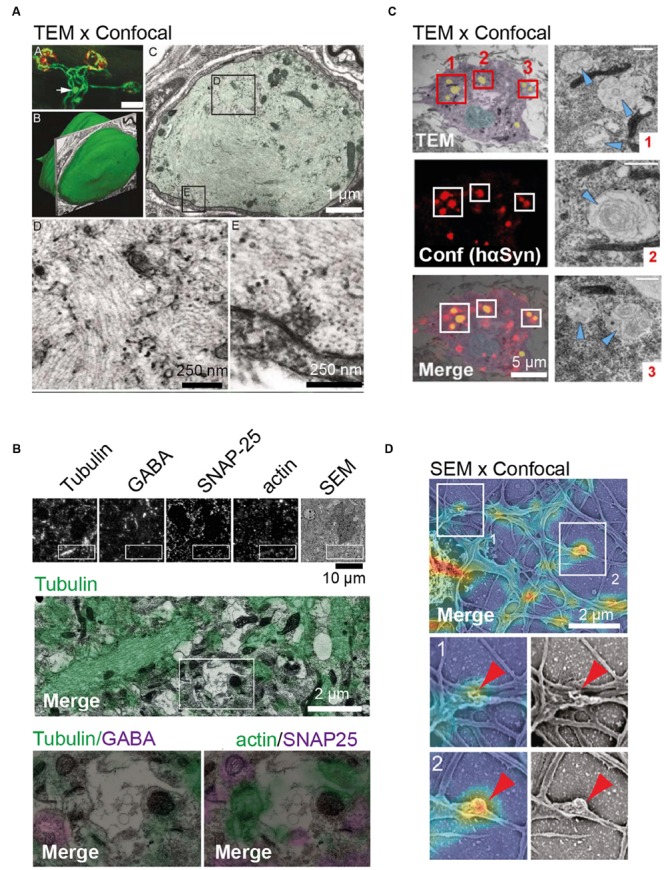
**Examples of *in situ* and *in vitro* correlative light electron microscopy (CLEM) approaches. (A)** *In situ* CLEM of confocal and transmission electron microscopy (TEM) images showing axonal retreat from neuromuscular junctions. Top left image shows confocal image depicting axonal bulb (arrow) present 25 um from the neuromuscular junction site (in red). Middle left image illustrating surface rendering of the bulb indicated above. Top right image shows TEM, shows that the bulb is sheathed by Schwann cells. TEM images at the bottom depict neurofilament disorganization in axonal bulb. **(B)** *In situ* CLEM of confocal and scanning electron microscopy (SEM) images of 70 nm section from the mouse cerebral cortex. From left to right: immunostaining of ultrathin sections for tubulin, GABA, SNAP-25, β-actin, and SEM image. Below, the boxed region is shown at a higher magnification. **(C)** *In vitro* CLEM of confocal and TEM images of hippocampal neurons. Ultrastructure of hα-Syn inclusions in cultured neurons from SNCA^+/-^ mice shown in TEM (top), confocal image (middle), and as merged CLEM image (bottom). For illustration, the nucleus is rendered in blue, the cytosol in purple, and inclusions in yellow. To the right, high-resolution images of inclusions (red boxes 1–3), with arrowheads indicating filamentous structures, are shown. **(D)** *In vitro* CLEM of confocal and SEM images of cultured hippocampal neurons. Alignment of SEM and fluorescence signal for actin in cultured hippocampal neurons (top) and magnified sections of actin-rich convoluted nodes that form along dendritic arbors (bottom) are shown. Scale bars: **(A)** 25 μm at the top left, 1 μm at the top right, 0.25 μm at the bottom; **(B)** 10 μm at the top and 2 μm at the bottom; **(C)** 5 μm; **(D)** 2 μm. Pictures reprinted with permission from: **(A)** (Bishop et al., 2004); **(B)** (Micheva and Smith, 2007); **(C)** (Fares et al., 2016); **(D)** (Galic et al., 2014).

To fully understand neuronal or synaptic function, the cellular context needs to be considered. It is thus not surprising, that large efforts have been put into elucidating ultrastructural information within the intact tissue. *In situ* CLEM, where data from life-cell fluorescence imaging is combined with EM micrographs, has advanced our understanding of neuronal circuits (Bock et al., 2011; Briggman et al., 2011; Lee et al., 2016), and has also shed light on subcellular behaviors such as axosome shedding (**Figure 1A**) (Bishop et al., 2004). An alternative strategy, frequently applied to gain information of individual neurons *in situ*, relies on array tomography (AT), where plastic-embedded tissue samples are sliced with an ultra-microtome, bonded array-wise onto a glass coverslip, stained, and finally imaged by fluorescence and electron microscopy (**Figure 1B**) (Micheva and Smith, 2007; Rah et al., 2013; Collman et al., 2015). By combining super-resolution techniques and serial sectioning, it was even possible to detect presynaptic dense projection proteins at a lateral resolution of 35–65 nm (Watanabe et al., 2011). While serial sectioning-based CLEM approaches allow studying biological samples with high axial and lateral resolution, sample preparation, and 3D image alignment can be challenging (Micheva and Smith, 2007; Collman et al., 2015). Here, SBEM and FIB-SEM based approaches, where *en-block* EM imaging and fluorescence approaches are merged to obtain CLEM images, have helped to streamline *in situ* studies focused on the complexity of the nervous system (Briggman et al., 2011; Blazquez-Llorca et al., 2015), or to reconstruct whole cortical neurons (Maco et al., 2013) and single synapses (Bosch et al., 2015).

Orthogonal to *in situ* approaches described above, CLEM can also be applied to neurons isolated from brain tissues and cultured *in vitro* (Banker and Cowan, 1977). As such, cultured neurons are compatible with each CLEM technique described above (**Figure 1C**) (Al Jord et al., 2014; Paez-Segala et al., 2015; Fares et al., 2016). Although these cells lack the physiological context usually encountered by neurons within a tissue, cultured neurons may still yield advantages compared to *in situ* samples depending on the biological question. One such example, and complementing the section-based CLEM assays described so far, is the analysis of processes at the cellular surface, such as curvature-dependent protein recruitment to deforming plasma membranes (**Figure 1D**) (Galic et al., 2014).

## Technical Considerations

In this section, we will focus on technical possibilities and limitations that need to be taken into consideration when designing a CLEM experiment. We start by discussing challenges arising from sample preparation. Next, we will survey what markers are suitable for which technique, and discuss strategies for alignment of corresponding fluorescence images and electron micrographs with respect to their potential and drawbacks.

### Sample Preparation for CLEM Images

The quality of correlative image alignment critically relies on the ability to maintain the native organization of the cell during fixation and subsequent sample preparation. Thus, CLEM techniques not only need to be optimized for signal strength, but also for shape preservation. Formaldehyde, glutaraldehyde, and osmium tetroxide (OsO_4_), have been the standard fixatives for decades, including for brain tissues that are due to their softness easily damaged during fixation and subsequent processing. Classical fixation protocols include whole animal perfusion via the vascular system for larger specimens (e.g., whole brain) as well as immersion of tissue slice and cultured cells. Once the biological sample is fixed, the actual preparation for the respective EM technique is rendered. For slice-based assays, two different approaches exist: the pre-embedding method and the post-embedding method. For both methods, correlative approaches have been reported (Watanabe et al., 2011; Kopek et al., 2012). In the pre-embedding method, the antigen–antibody reaction is performed before (i.e., pre) plastic embedding and subsequent ultrathin sectioning. While better for preserving ultra-structures, this approach is often hindered by poor penetration of the antibody. In the post-embedding method, the antigen–antibody reaction is performed after (i.e., post) plastic embedding. As labeling takes place on thin tissue slices, antigens are more easily accessible. However, OsO_4_, which is often used as fixative and stain for membrane structures, can quench the fluorescence signal, and epoxy-based resins used due to its good preservation and ultra-sectioning properties partially inhibit photo-switching of fluorophores (Kim et al., 2015). Here, a number of modifications, such as using acrylic resins as embedding material or replacing OsO_4_ with uranyl acetate, tannic acid or *p*-phenylenediamine, have helped to enhance compatibility and immunoreactivity (Phend et al., 1995; Osamura et al., 2000; Akagi et al., 2006; Kim et al., 2015). Complementing these slice-based assays, cells can also be prepared for analysis of surface features. As before, samples are fixed. However, rather than embedding the sample, a platinum replica of the surface is generated (Heuser et al., 1976), or the sample is subjected to critical point drying and surface staining, prior to image acquisition via SEM. Finally, one last concern is that chemical fixation is not instantaneous (Szczesny et al., 1996). To circumvent this issue, quick-freezing, where samples are ‘slammed’ on a super-cold block of metal sprayed with liquid helium (Heuser et al., 1976) or high-pressure freezing, where samples are frozen in milli seconds while pressure is increased to avoid water crystallization (Hunziker et al., 1984; Dahl and Staehelin, 1989), have improved preservation quality.

### Picking the Right Markers for CLEM

**FIGURE 2:**
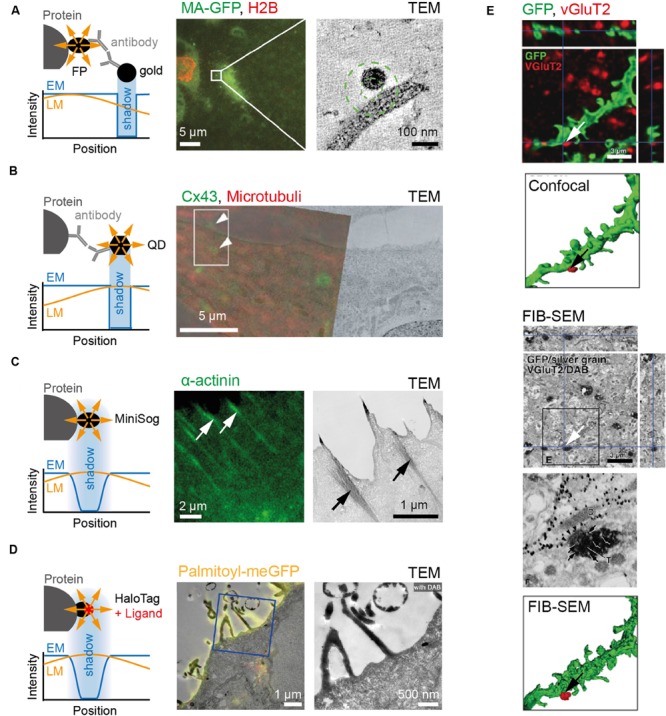
**Examples of markers commonly used for CLEM. (A)** To the left, cartoon depicting immune-gold labeling of genetically encoded fluorescent protein. Note difference between the relative position of signal in fluorescence (yellow) and electron microscope (blue) images caused by antibodies. To the right, an example showing fluorescence and electron micrographs of HIV particle labeled with MA-EGFP on MDCK cells expressing RFP-tagged Histone 2B. **(B)** To the left, cartoon depicting protein and signal from QD in fluorescence (yellow) and electron microscope (blue) images. Note difference between protein epitope recognized by antibody and QD signal position. To the right, example depicting RFL6 fibroblasts fixed and stained with primary antibodies followed by secondary antibodies linked to QDs. QDs identify Cx43 at gap junctions and trafficking intermediates (green) and α-tubulin in microtubules (red). **(C)** To the left, cartoon depicting genetically encoded MiniSOG, as well as the relative position and signal shape for fluorescence (yellow) and electron microscope (blue) images. To the right, an example showing fluorescence and electron micrographs of HeLa cells expressing miniSOG labeled α-actinin. **(D)** To the left, cartoon depicting HaloTag labeling of protein as well as position and signal shape for fluorescence (yellow) and electron microscope (blue) images. To the right, an example showing fluorescence and electron micrographs of Hela cell transfected with Palmitoyl-HaloTag-meGFP. **(E)** Dendrites of medium-size spiny neurons in the rat neostriatum labeled with membrane-targeted GFP and immunolabeled with Cy5 against vesicular glutamate transporter2 (VGluT2; top). After detection by fluorescence microscopy, GFP and VGluT2 immunoreactivities were further developed for focused ion-beam SEM (FIB-SEM) via immunogold/silver enhancement and immunoperoxidase/DAB methods, respectively (bottom). Scale bars: **(A)** 5 μm to the left and 100 nm to the right; **(B)** 5 μm; **(C)** 2 μm to the left and 1 μm to the right; **(D)** 1 μm to the left and 500 nm to the right; **(E)** 3 μm. Pictures reprinted with permission from: **(A)** (Kukulski et al., 2011); **(B)** (Giepmans et al., 2005); **(C)** (Shu et al., 2011); **(D)** (Liss et al., 2015); **(E)** (Sonomura et al., 2013).

Unfortunately, not all markers are equally well suited for individual CLEM approaches. To account for these limitations, a series of labeling techniques has emerged. Considering its widespread availability, the most commonly used markers for CLEM are genetically encoded fluorescence tags, such as GFP, that can be detected in electron micrographs via immuno-gold labeling (**Figure 2A**) (Kukulski et al., 2011; Galic et al., 2012). In order to distinguish several proteins in the same sample, immuno-gold particles of variable sizes directed against different fluorescence tags can be used. However, since sample fixation may interfere with genetically encoded fluorescence markers (Muller-Reichert and Verkade, 2014) and gold particles may quench the fluorophore in parallel fluorescence/electron imaging (Kandela and Albrecht, 2007), additional markers are desirable. One such alternative relies on quantum dots (QDs; **Figure 2B**), which were shown to have a 10 times higher labeling efficiency than immuno-gold (Giepmans et al., 2005; Kuipers et al., 2015). These semiconductor nanocrystals consist of a cadmium selenide core surrounded by a zinc sulfide shell coated with affinity ligands (e.g., antibodies) for targeting the desired biomolecules (Alivisatos, 1996). Intriguingly, as the fluorescence wavelength of these photo-stable nanocrystals depends on their core size, it is possible to label samples with fluorescently different QDs, and then to distinguish individual QDs in electron micrographs by size (Giepmans et al., 2005; Kuipers et al., 2015). A minor disadvantage of QDs, however, lies in the difficulty to precisely measure sizes of small QDs due to its low contrast in EM (Brown and Verkade, 2010). While widely used, both methods are hampered by a localization error due to spatial separation between the protein epitope and the electro-dense particle that is visualized in light and electron micrographs (Kandela et al., 2007; van Donselaar et al., 2007). To bypass this imprecision, Aptamers directed against the fluorescence tag (Shui et al., 2012) linked to gold (Javier et al., 2008; Chang et al., 2013), or nano-bodies directed against fluorescent proteins that can be conjugated to gold nanoparticles (Van de Broek et al., 2011; Ries et al., 2012) may provide promising alternatives.

Orthogonal to these approaches, several strategies have emerged that take advantage of singlet oxygen generators. Genetically encoded singlet oxygen generators are of particular relevance for samples that are investigated ‘*en block*’ (i.e., SBEM and FIB-SEM) and thus preclude post-staining of individual sections. One such example is the genetically encoded miniSOG (**Figure 2C**), which not only fluoresces when illuminated by blue light, but also yields an osmophilic reaction product by catalyzing the polymerization of 3,3′-Diaminobenzidine (DAB) into electron-dense polymers that are detectable in electron micrographs (Shu et al., 2011). The same strategy is also applicable using the Halo-Tag system, where a modified haloalkene dehalogenase (Los et al., 2008) is used to covalently bind to specific ligands such as tetramethylrhodamine (TMR; **Figure 2D**). Like miniSOG, TMR fluoresces upon illumination and is visible in EM due to DAB oxidation (Liss et al., 2015). Other self-labeling systems are provided by SNAP/CLIP-tags, in which the enzyme domain is bound to the protein of interest and labeled with a cell–permeable fluorescent ligand (Liss et al., 2015). Finally, cells can also be transfected with APEX, a non-fluorescent peroxidase that withstands strong fixation to yield light-independently EM contrast (Martell et al., 2012). However, considering that the reaction product can diffuse, these approaches may yield best results when staining enclosed structures. Notably, multiple procedures can be combined within a sample for visualization (**Figure 2E**).

### Strategies of Aligning Fluorescence Image and Electron Micrograph in CLEM

Correlation of fluorescence images and electron micrographs can be challenging for a number of reasons. For one, the region of interest can cover less than 0.0004% of the total sample area (Begemann et al., 2015), thus offering only on a limited number of reference points for navigation. And even after the region of interest acquired at the light microscope is found again on the electron microscope, rotation, magnification, and tilting angle still need to be adjusted for image alignment. This is of particular relevance in pre-embedding approaches, where, unlike to post-embedding strategies, the angle of the respective acquisition planes may differ. To tackle these obstacles, several strategies for navigation and image alignment have emerged for *in situ* and *in vitro* approaches.

For *in situ* CLEM, various fiduciary markers have been described that are equally suitable for pre-embedding and post-embedding strategies. In both approaches, the region of interest in the embedded sample is identified using a previously determined set of reference points. Here both, biological (e.g., blood vessels and dirt) and artificial (e.g., silica beads and gold markers) landmarks have proven to be useful for navigation. Once identified, individual EM sections are made and, if desired, 3D reconstruction of individual slices can be prepared by manual or automatic alignment using fiduciary landmarks present in adjacent sections (Bishop et al., 2004; Bock et al., 2011; Lee et al., 2016). Finally, electron micrographs (2D or 3D) are merged with the corresponding fluorescence images either manually or automatically using large features visible in both images (e.g., blood vessels and silica beads) for coarse alignment. For subsequent fine-adjustment, subcellular structures (e.g., gold fiduciary markers, nucleus, and filopodia) can be used. To estimate how accurate the alignment is, cross-correlation analysis between light and electron micrographs can be performed.

*In vitro* CLEM approaches permit the generation of additional reference systems for image alignment on the plating substrate (i.e., glass and plastic). For example, a unique reference point can be created at the position where the fluorescence image is acquired, using for instance laser etching (Colombelli et al., 2008; Bishop et al., 2011; Urwyler et al., 2015) or a scratching device such as diamond objective markers (Sochacki et al., 2014). Upon processing, this reference point can then be used for navigation on the electron microscope and for subsequent image alignment. Another popular reference system relies on pre-formed structured (Svitkina, 2009; Benedetti et al., 2014) and stochastic (Begemann et al., 2015) micro-patterns or fiducial landmarks (Kukulski et al., 2011) for re-finding the region of interest on the EM as well as subsequent image alignment. Intriguingly, such reference points are not only suitable for manual alignment, but also for software-assisted solutions (Begemann et al., 2015), rendering these approaches an attractive entry point compared to more sophisticated CLEM strategies.

Finally, simultaneous fluorescence and electron imaging may provide an alternative strategy. Here, a number of custom-made (Liv et al., 2013; Nishiyama et al., 2014; Peddie et al., 2014; de Boer et al., 2015) and commercial (e.g., from Fei and Zeiss) instruments have been presented. These instruments, which are equally suitable for *in situ* and *in vitro* samples, integrate light- and electron imaging in one apparatus (iCLEM), thus ensuring dual imaging of the region of interest without need of later image alignment. However, as sample preparation for iCLEM is restricted to techniques suitable for parallel LM and EM (Agronskaia et al., 2008), and due to the limited availability of such instruments, iCLEM has not yet reached the optional users.

## CLEM–Potential and Limitations

**FIGURE 3:**
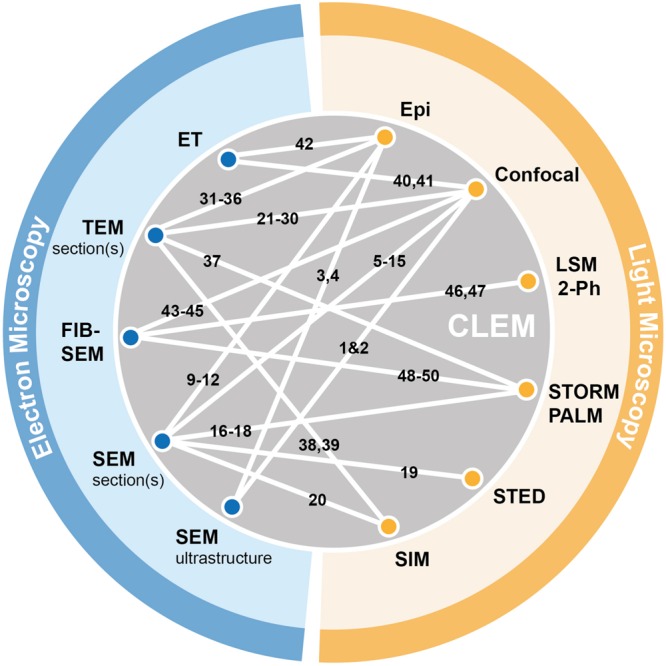
**Graphical summary of existing CLEM approaches.** Electron microscopy (blue) and light-based techniques (yellow) that were successfully combined in correlative approaches are depicted by white lines connecting the respective methods. References corresponding to individual publications using specific CLEM approaches (numbered 1–50) can be found in the Supplementary Materials (Supplementary Table S1).

In this review, we have discussed possible combinations for correlating optical and electron microscopy approaches. We mentioned how improvements in imaging techniques, sample preparation, markers, and image alignment have facilitated development of novel CLEM approaches, and showed examples where these strategies were successfully used for structure-function analysis in neurons and synapses. In order to visualize the combinatorial potential, we provide a chart summarizing published CLEM pairings (**Figure 3**).

While correlative approaches have revealed aspects of neuronal and synaptic function that would not have been possible without it, challenges still remain due to the limited temporal and spatial resolution of CLEM. From this perspective, recent work on liquid cell electron microscopy (Ross, 2015), where electron-permeable membranes are used to host aqueous solutions under atmospheric pressure conditions for TEM and SEM imaging (Danilatos et al., 2011; Ominami et al., 2015), cryo-ET to study the supramolecular architecture of cellular structures (Medalia et al., 2002; Beck et al., 2007), and the continuous increase in sophistication and computational power used for image analysis have captured the imagination of people. While it remains elusive whether these and other emerging techniques will merge or replace CLEM-based studies, they are exemplary for an ongoing push toward visualization and comprehensive analysis of biological function on the structural level.

Taken together, the possibilities of applying correlative approaches to study neuronal and synapse function are growing thanks to the continuous progress in techniques, tools and protocols for combining these two types of microscopy. Alas, while correlative approaches in neurobiology have substantially increased in quality and availability, truly artifact-free preparation techniques for precise molecule-localization are yet to be achieved.

## Author Contributions

IB and MG conceived the idea, prepared illustrations for publication and wrote the manuscript.

## Conflict of Interest Statement

The authors declare that the research was conducted in the absence of any commercial or financial relationships that could be construed as a potential conflict of interest.
