# Supplementary material for: Correlative Light Electron Microscopy: Connecting Synaptic Structure and Function
Source: Front Synaptic Neurosci. 2016 Aug 23;8:28. doi: 10.3389/fnsyn.2016.00028 (PMC4993758; doi:10.3389/fnsyn.2016.00028)
Supplement: Supplementary file 1 [file Table_1.PDF]

Supplemental Table 1: References for Fig. 3

Correlative Light Electron Microscopy: Connecting Synaptic Structure and Function

Isabell Begemann[1,2], Milos Galic[1,2,*]

[1] DFG Cluster of Excellence 'Cells in Motion', (EXC 1003), University of Muenster
Waldeyerstrasse 15, 48149 Muenster, Germany

[2] Institute of Medical Physics and Biophysics, University of Muenster
Robert Koch Strasse 31, 48149 Muenster, Germany

Supplemental Table 1: References for Figure 3

	#	Reference
**SEM (ultrastruct.)- Confocal**	**1**	Begemann, I., A. Viplav, C. Rasch and M. Galic (2015). "Stochastic Micro-Pattern for Automated Correlative Fluorescence - Scanning Electron Microscopy." Sci Rep **5**: 17973.
	**2**	Galic, M., F. C. Tsai, S. R. Collins, M. Matis, S. Bandara and T. Meyer (2014). "Dynamic recruitment of the curvature-sensitive protein ArhGAP44 to nanoscale membrane deformations limits exploratory filopodia initiation in neurons." Elife **3**.
**SEM (ultrastruct.) - EPI**	**3**	Begemann, I., A. Viplav, C. Rasch and M. Galic (2015). "Stochastic Micro-Pattern for Automated Correlative Fluorescence - Scanning Electron Microscopy." Sci Rep **5**: 17973.
	**4**	Liv, N., A. C. Zonnevylle, A. C. Narvaez, A. P. Effting, P. W. Voorneveld, M. S. Lucas, J. C. Hardwick, R. A. Wepf, P. Kruit and J. P. Hoogenboom (2013). "Simultaneous correlative scanning electron and high-NA fluorescence microscopy." PLoS One **8**(2): e55707.
**SEM (sections) - Confocal**	**5**	Micheva, K. D. and S. J. Smith (2007). "Array tomography: a new tool for imaging the molecular architecture and ultrastructure of neural circuits." Neuron **55**(1): 25-36.
	**6**	Shu, X., V. Lev-Ram, T. J. Deerinck, Y. Qi, E. B. Ramko, M. W. Davidson, Y. Jin, M. H. Ellisman and R. Y. Tsien (2011). "A genetically encoded tag for correlated light and electron microscopy of intact cells, tissues, and organisms." PLoS Biol **9**(4): e1001041.
	**7**	Watanabe, S., A. Punge, G. Hollopeter, K. I. Willig, R. J. Hobson, M. W. Davis, S. W. Hell and E. M. Jorgensen (2011). "Protein localization in electron micrographs using fluorescence nanoscopy." Nature Methods **8**(1): 80-U117.
	**8**	Urwyler, O., A. Izadifar, D. Dascenco, M. Petrovic, H. H. He, D. Ayaz, A. Kremer, S. Lippens, P. Baatsen, C. J. Guerin and D. Schmucker (2015). "Investigating CNS synaptogenesis at single-synapse resolution by combining reverse genetics with correlative light and electron microscopy." Development **142**(2): 394-405.
**SEM (sections) - EPI**	**9**	Collman, F., J. Buchanan, K. D. Phend, K. D. Micheva, R. J. Weinberg and S. J. Smith (2015). "Mapping Synapses by Conjugate Light-Electron Array Tomography." Journal of Neuroscience **35**(14): 5792-5807.
	**10**	Micheva, K. D. and S. J. Smith (2007). "Array tomography: a new tool for imaging the molecular architecture and ultrastructure of neural circuits." Neuron **55**(1): 25-36.
	**11**	Liv, N., A. C. Zonnevylle, A. C. Narvaez, A. P. Effting, P. W. Voorneveld, M. S. Lucas, J. C. Hardwick, R. A. Wepf, P. Kruit and J. P. Hoogenboom (2013). "Simultaneous correlative scanning electron and high-NA fluorescence microscopy." PLoS One **8**(2): e55707.
	**12**	Peddie, C. J., K. Blight, E. Wilson, C. Melia, J. Marrison, R. Carzaniga, M. C. Domart, P. O'Toole, B. Larijani and L. M. Collinson (2014). "Correlative and integrated light and electron microscopy of in-resin GFP fluorescence, used to localise diacylglycerol in mammalian cells." Ultramicroscopy **143**: 3-14.
**SEM (sections) - AT**	**13**	Collman, F., J. Buchanan, K. D. Phend, K. D. Micheva, R. J. Weinberg and S. J. Smith (2015). "Mapping Synapses by Conjugate Light-Electron Array Tomography." Journal of Neuroscience **35**(14): 5792-5807.
	**14**	Micheva, K. D. and S. J. Smith (2007). "Array tomography: a new tool for imaging the molecular architecture and ultrastructure of neural circuits." Neuron **55**(1): 25-36.
	**15**	Shu, X., V. Lev-Ram, T. J. Deerinck, Y. Qi, E. B. Ramko, M. W. Davidson, Y. Jin, M. H. Ellisman and R. Y. Tsien (2011). "A genetically encoded tag for correlated light and electron microscopy of intact cells, tissues, and organisms." PLoS Biol **9**(4): e1001041.

**SEM (sections) – PALM/STORM**	**16**	Kopek, B. G., G. Shtengel, J. B. Grimm, D. A. Clayton and H. F. Hess (2013). "Correlative photoactivated localization and scanning electron microscopy." PLoS One **8**(10): e77209.
	**17**	Paez-Segala, M. G., M. G. Sun, G. Shtengel, S. Viswanathan, M. A. Baird, J. J. Macklin, R. Patel, J. R. Allen, E. S. Howe, G. Piszczek, H. F. Hess, M. W. Davidson, Y. Wang and L. L. Looger (2015). "Fixation-resistant photoactivatable fluorescent proteins for CLEM." Nat Methods **12**(3): 215-218, 214 p following 218.
	**18**	Watanabe, S., A. Punge, G. Hollopeter, K. I. Willig, R. J. Hobson, M. W. Davis, S. W. Hell and E. M. Jorgensen (2011). "Protein localization in electron micrographs using fluorescence nanoscopy." Nature Methods **8**(1): 80-U117.
**SEM (sections) - STED**	**19**	Watanabe, S., A. Punge, G. Hollopeter, K. I. Willig, R. J. Hobson, M. W. Davis, S. W. Hell and E. M. Jorgensen (2011). "Protein localization in electron micrographs using fluorescence nanoscopy." Nature Methods **8**(1): 80-U117.
**SEM (sections) - SIM**	**20**	Wanner, G., E. Schroeder-Reiter, W. Ma, A. Houben and V. Schubert (2015). "The ultrastructure of mono- and holocentric plant centromeres: an immunological investigation by structured illumination microscopy and scanning electron microscopy." Chromosoma **124**(4): 503-517.
**TEM – Confocal**	**21**	Ligeon, L. A., N. Barois, E. Werkmeister, A. Bongiovanni and F. Lafont (2015). "Structured illumination microscopy and correlative microscopy to study autophagy." Methods **75**: 61-68.
	**22**	Deerinck, T. J., M. E. Martone, V. Levram, D. P. L. Green, R. Y. Tsien, D. L. Spector, S. Huang and M. H. Ellisman (1994). "Fluorescence Photooxidation with Eosin - a Method for High-Resolution Immunolocalization and in-Situ Hybridization Detection for Light and Electron-Microscopy." Journal of Cell Biology **126**(4): 901-910.
	**23**	Giepmans, B. N., T. J. Deerinck, B. L. Smarr, Y. Z. Jones and M. H. Ellisman (2005). "Correlated light and electron microscopic imaging of multiple endogenous proteins using Quantum dots." Nat Methods **2**(10): 743-749.
	**24**	Hohensee, S., W. Bleiss and C. Duch (2008). "Correlative electron and confocal microscopy assessment of synapse localization in the central nervous system of an insect." J Neurosci Methods **168**(1): 64-70.
	**25**	Modla, S., J. Mendonca, K. J. Czymmek and R. E. Akins (2010). "Identification of neuromuscular junctions by correlative confocal and transmission electron microscopy." J Neurosci Methods **191**(2): 158-165.
	**26**	Martell, J. D., Deerinck, T. J., Sancak, Y., Poulos, T. L., Mootha, V. K., Sosinsky, G. E., Ellisman, M. H. and Ting, A. Y. (2012): "Engineered ascorbate peroxidase as a genetically encoded reporter for electron microscopy". Nat Biotechnol **30**(11): 1143-8.
	**27**	Shu, X., V. Lev-Ram, T. J. Deerinck, Y. Qi, E. B. Ramko, M. W. Davidson, Y. Jin, M. H. Ellisman and R. Y. Tsien (2011). "A genetically encoded tag for correlated light and electron microscopy of intact cells, tissues, and organisms." PLoS Biol **9**(4): e1001041.
	**28**	Urwyler, O., A. Izadifar, D. Dascenco, M. Petrovic, H. H. He, D. Ayaz, A. Kremer, S. Lippens, P. Baatsen, C. J. Guerin and D. Schmucker (2015). "Investigating CNS synaptogenesis at single-synapse resolution by combining reverse genetics with correlative light and electron microscopy." Development **142**(2): 394-405.
	**29**	Vicidomini, G., M. C. Gagliani, K. Cortese, J. Krieger, P. Buescher, P. Bianchini, P. Boccacci, C. Tacchetti and A. Diaspro (2010). "A novel approach for correlative light electron microscopy analysis." Microsc Res Tech **73**(3): 215-224.

**TEM - AT**	**30**	Collman, F., J. Buchanan, K. D. Phend, K. D. Micheva, R. J. Weinberg and S. J. Smith (2015). "Mapping Synapses by Conjugate Light-Electron Array Tomography." Journal of Neuroscience **35**(14): 5792-5807.
**TEM - EPI**	**31**	Collman, F., J. Buchanan, K. D. Phend, K. D. Micheva, R. J. Weinberg and S. J. Smith (2015). "Mapping Synapses by Conjugate Light-Electron Array Tomography." Journal of Neuroscience **35**(14): 5792-5807.
	**32**	Deerinck, T. J., M. E. Martone, V. Levram, D. P. L. Green, R. Y. Tsien, D. L. Spector, S. Huang and M. H. Ellisman (1994). "Fluorescence Photooxidation with Eosin - a Method for High-Resolution Immunolocalization and in-Situ Hybridization Detection for Light and Electron-Microscopy." Journal of Cell Biology **126**(4): 901-910.
	**33**	Giepmans, B. N., T. J. Deerinck, B. L. Smarr, Y. Z. Jones and M. H. Ellisman (2005). "Correlated light and electron microscopic imaging of multiple endogenous proteins using Quantum dots." Nat Methods **2**(10): 743-749.
	**34**	Kandela, I. K., R. Bleher and R. M. Albrecht (2007). "Multiple correlative immunolabeling for light and electron microscopy using fluorophores and colloidal metal particles." J Histochem Cytochem **55**(10): 983-990.
	**35**	Korobova, F. and T. Svitkina (2010). "Molecular Architecture of Synaptic Actin Cytoskeleton in Hippocampal Neurons Reveals a Mechanism of Dendritic Spine Morphogenesis." Molecular Biology of the Cell **21**(1): 165-176.
	**36**	Peddie, C. J., K. Blight, E. Wilson, C. Melia, J. Marrison, R. Carzaniga, M. C. Domart, P. O'Toole, B. Larijani and L. M. Collinson (2014). "Correlative and integrated light and electron microscopy of in-resin GFP fluorescence, used to localise diacylglycerol in mammalian cells." Ultramicroscopy **143**: 3-14.
**TEM – STORM/ PALM**	**37**	Paez-Segala, M. G., M. G. Sun, G. Shtengel, S. Viswanathan, M. A. Baird, J. J. Macklin, R. Patel, J. R. Allen, E. S. Howe, G. Piszczek, H. F. Hess, M. W. Davidson, Y. Wang and L. L. Looger (2015). "Fixation-resistant photoactivatable fluorescent proteins for CLEM." Nat Methods **12**(3): 215-218, 214 p following 218.
**TEM - SIM**	**38**	Al Jord, A., A. I. Lemaitre, N. Delgehyr, M. Faucourt, N. Spassky and A. Meunier (2014). "Centriole amplification by mother and daughter centrioles differs in multiciliated cells." Nature **516**(7529): 104-107.
	**39**	Ligeon, L. A., N. Barois, E. Werkmeister, A. Bongiovanni and F. Lafont (2015). "Structured illumination microscopy and correlative microscopy to study autophagy." Methods **75**: 61-68.
**ET - Confocal**	**40**	Shu, X., V. Lev-Ram, T. J. Deerinck, Y. Qi, E. B. Ramko, M. W. Davidson, Y. Jin, M. H. Ellisman and R. Y. Tsien (2011). "A genetically encoded tag for correlated light and electron microscopy of intact cells, tissues, and organisms." PLoS Biol **9**(4): e1001041.
	**41**	Vicidomini, G., M. C. Gagliani, K. Cortese, J. Krieger, P. Buescher, P. Bianchini, P. Boccacci, C. Tacchetti and A. Diaspro (2010). "A novel approach for correlative light electron microscopy analysis." Microsc Res Tech **73**(3): 215-224.
**ET – EPI**	**42**	Grabenbauer, M., W. J. C. Geerts, J. Fernadez-Rodriguez, A. Hoenger, A. J. Koster and T. Nilsson (2005). "Correlative microscopy and electron tomography of GFP through photooxidation." Nature Methods **2**(11): 857-862.
**FIB-SEM – Confocal**	**43**	Sonomura, T., T. Furuta, I. Nakatani, Y. Yamamoto, S. Honma and T. Kaneko (2014). "Attempt of correlative observation of morphological synaptic connectivity by combining confocal laser-scanning microscope and FIB-SEM for immunohistochemical staining technique." Microscopy (Oxf) **63 Suppl 1**: i8.

	**44**	Kopek, B. G., G. Shtengel, J. B. Grimm, D. A. Clayton and H. F. Hess (2013). "Correlative photoactivated localization and scanning electron microscopy." PLoS One **8**(10): e77209.
	**45**	Canty, A. J., L. Huang, J. S. Jackson, G. E. Little, G. Knott, B. Maco and V. De Paola (2013). "In-vivo single neuron axotomy triggers axon regeneration to restore synaptic density in specific cortical circuits." Nat Commun 4: 2038.
**FIB-SEM – PALM/STORM**	**46**	Kopek, B. G., G. Shtengel, C. S. Xu, D. A. Clayton and H. F. Hess (2012). "Correlative 3D superresolution fluorescence and electron microscopy reveal the relationship of mitochondrial nucleoids to membranes." Proc Natl Acad Sci U S A **109**(16): 6136-6141.
	**47**	Wanner, G., E. Schroeder-Reiter, W. Ma, A. Houben and V. Schubert (2015). "The ultrastructure of mono- and holocentric plant centromeres: an immunological investigation by structured illumination microscopy and scanning electron microscopy." Chromosoma **124**(4): 503-517.
**FIB-SEM – 2 Photon/LSM**	**48**	Blazquez-Llorca, L., E. Hummel, H. Zimmerman, C. Zou, S. Burgold, J. Rietdorf and J. Herms (2015). "Correlation of two-photon in vivo imaging and FIB/SEM microscopy." J Microsc **259**(2): 129-136.
	**49**	Maco, B., Cantoni, M., Holtmaat, A., Kreshuk, A., Hamprecht, F. A., and Knott, G. W. (2014). Semiautomated correlative 3D electron microscopy of *in vivo*-imaged axons and dendrites. *Nat. Protoc*. 9:1354–1366. doi: 10.1038/nprot.2014.101
	**50**	Canty, A. J., L. Huang, J. S. Jackson, G. E. Little, G. Knott, B. Maco and V. De Paola (2013). "In-vivo single neuron axotomy triggers axon regeneration to restore synaptic density in specific cortical circuits." Nat Commun **4**: 2038.
